# Induction Therapy Followed by Surgery for Unresectable Thymic Epithelial Tumours

**DOI:** 10.3389/fonc.2021.791647

**Published:** 2022-01-05

**Authors:** Shuai Wang, Jiahao Jiang, Jian Gao, Gang Chen, Yue Fan, Bei Xu, Jihong Dong, Shisuo Du, Junzhen Liu, Jianyong Ding

**Affiliations:** ^1^Department of Thoracic Surgery, Zhongshan Hospital, Fudan University, Shanghai, China; ^2^Department of Pathology, Zhongshan Hospital, Fudan University, Shanghai, China; ^3^Department of Integrated Traditional Chinese Medicine (TCM) & Western Medicine, Zhongshan Hospital, Fudan University, Shanghai, China; ^4^Department of Medical Oncology, Zhongshan Hospital, Fudan University, Shanghai, China; ^5^Department of Neurology, Zhongshan Hospital, Fudan University, Shanghai, China; ^6^Department of Radiotherapy, Zhongshan Hospital, Fudan University, Shanghai, China; ^7^Department of Radiology, Zhongshan Hospital, Fudan University, Shanghai, China

**Keywords:** thymic epithelial tumors, survival, induction therapy, complete resection, stage

## Abstract

**Background and Objectives:**

The treatment of unresectable thymic epithelial tumours (TETs) remains controversial. Here, we present the efficacy and safety of induction therapy followed by surgery for unresectable TET.

**Methods:**

Eighty-one patients with unresectable TETs treated with induction therapy followed by surgery were selected from a retrospective review of consecutive TETs from January 2005 to January 2021. Clinicopathological data were analyzed to assess tumour responses, resectability, adverse events, progression-free survival (PFS) and overall survival (OS).

**Results:**

Induction therapy produced a major tumour response rate of 69.1%, a tumour response grade (TRG) 1-3 rate of 84.0% and an R0 resection rate of 74.1%. The most common toxic effects were all-grade neutropenia (35.8%) and anaemia (34.6%). The 10-year OS and PFS rates were 45.7% and 35.2%. Multivariate analysis showed that ypTNM stage, ypMasaoka stage, complete resection, and TRG were significant independent prognostic factors. Exploratory research revealed that different induction modalities and downstaging of T, N, M, TNM, or Masaoka classifications did not significantly alter the pooled hazard ratio for survival.

**Conclusions:**

Induction therapy followed by surgery is well tolerated in patients with unresectable TETs, with encouraging R0 resection rates. Multimodality management provides good control of tumors for unresectable TET patients.

## Highlights

- Induction therapy followed by surgery had encouraging tumor response and complete resection rate for initially unresectable thymic epithelial tumors.
- ypTNM stage, ypMasaoka stage, complete resection, and tumor response grade (TRG) were all independent significant prognostic factors.
- Induction therapy followed by surgery provides good control of tumor for unresectable TETs patients.

## Introduction

Thymic epithelial tumours (TETs), comprised of thymoma and thymic carcinoma, are the most common tumours in the anterior mediastinum (1). However, a notable proportion of patients with TETs present at advanced stages based on the International Thymic Malignancy Interest Group (2). Until now, TETs have generally been considered surgical diseases, and complete resection remains the mainstay of treatment. However, complete resection is challenging and unavailable for advanced lesions due to invasion of the mediastinal structures or diffuse pleural implants. Successful treatment of unresectable TETs involves multidisciplinary evaluation. A variety of chemotherapy regimens for TET have been reported. A majority of patients with TETs in the reported studies were able to proceed to resection with clinical response rates of 62%-100% and complete resection rates of 22%-92% (3, 4). Preoperative radiation has been used in locally advanced malignancies, and this strategy has also been examined in TETs. However, there was no survival difference between patients who underwent preoperative radiation and those who did not based on retrospective studies (5). Loehrer PJ Sr. et al. (6) reported that preoperative chemoradiation had encouraging clinical responses and potentially high pathological response rates. The opportunity to achieve complete resection after induction therapy followed by surgery has led to interest in pursuing this strategy in unresectable TETs.

In general, previous studies on induction therapy mainly comprised small retrospective case series. Investigators were unable to draw firm conclusions about the outcomes of induction treatment. Because of the rarity of TETs and their natural behaviour, it is difficult to reach definite conclusions with small samples. Currently, the standard management of unresectable TETs remains controversial. We performed a study with multimodal treatments to improve the resectability and responses of unresectable TETs. The objective of the present study was to retrospectively evaluate the tumour responses and prognosis of patients with unresectable TETs treated with induction therapy followed by surgery.

## Methods

### Patients Selection

From January 2005 to January 2021, we selected patients with TETs who underwent induction therapy followed by surgery. Approval was obtained from the Research Ethics Committee of Zhongshan Hospital, Fudan University. Informed consent was obtained, and this study was compliant with the Helsinki Declaration. The inclusion criteria were as follows: (a) primary mediastinal TETs were confirmed by pathological examination according to the World Health Organization classification (7). (b) Patients received multidisciplinary consultation for mediastinal tumours, and unresectable advanced TET patients were treated with induction therapy followed by resection. Patients were deemed to have primary unresectable TETs as judged by a joint consensus of a multimodality oncology team (thoracic surgeon, medical oncologist, radiation oncologist, radiologist, and pathologist). Patients were judged to be unresectable with radiographic findings of extensive invasion, large tumors with indistinct borders, or great vessel invasion. Deep intrathoracic or cervical nodal involvement, pleural or pericardial nodules, and pulmonary intraparenchymal nodule or distant organ metastasis were also defined as poorly resectable. (c) Tumours were dimensionally measurable lesions according to the Response Evaluation Criteria in Solid Tumours (RECIST) criteria. We excluded patients with (a) incomplete information regarding pathological variables and clinical data, (b) noncurative resection (i.e., biopsy only), and (c) surgical resection for recurrence after primary treatment. Tumour stage was determined using the Masaoka staging system and the 8^th^ edition TNM classification of TETs (8, 9). Medical records and laboratory data were collected in accordance with the recommendation of the International Thymic Malignancy Interest Group (ITMIG) standard definitions and policies (10).

### Treatment and Evaluation

Patients with inoperable disease were treated with induction therapy, as judged by joint consensus of a multidisciplinary mediastinal oncology team (thoracic surgery, medical oncology, pathology, neurology and radiology). All patients had an initial biopsy that confirmed the suspected TETs before induction therapy. Induction therapies were quite heterogeneous and individualized depending on each patient’s status, including chemotherapy, radiotherapy or sequential/concurrent chemoradiation. Chemotherapies included different cycles of chemotherapy using cisplatin-based regimens of CAP (cyclophosphamide, doxorubicin, cisplatin), TP (paclitaxel, carboplatin/cisplatin), DP (docetaxel, cisplatin), VP (etoposide, cisplatin) or GP (gemcitabine, cisplatin). Radiotherapy was performed with involved fields that covered the primary and metastatic tumours with margins of approximately 1–2 cm. Three-dimensional conformal or intensity-modulated radiation with a total dose of 60–70 Gy was administered for radiation alone, and lower doses of 40–50 Gy were administered for sequential/concurrent chemoradiation. After completion of induction therapy, TETs were reevaluated and restaged by CT, MRI or PET scan. The tumour response to induction therapy was evaluated as progressive disease (PD), stable disease (SD), partial response (PR) or complete response (CR). Specimens were evaluated by independent senior pathologists. All postoperative specimens were reviewed for tumour response grade (TRG) based on the percent necrosis and viable tumour on the entire tumour bed as following: TRG1: no viable tumor; TRG2: rare residual viable tumor cells scattered through fibrosis; TRG3: increase in number of residual tumor cells, fibrosis predominant; TRG4: residual viable tumor outgrowing fibrosis; TRG5: no regressive changes (11). The Common Terminology Criteria for Adverse Events (CTCAE 3.0) was used to grade the severity of adverse events (AEs). Total thymectomy was performed with the goal of complete removal of the tumour and all attached structures. The operation procedure was reported partially in our previous study (12). Complete surgical resection (R0) was defined as en bloc resection of the tumour without macroscopic (R2) or microscopic (R1) residuals.

### Statistical Analysis

The chi-square test and Fisher’s exact test were used to compare categorical variables, and Student’s t-test was used to compare continuous variables. Overall survival (OS) was defined as the period from the date of treatment initiation to the date of death from any cause or the final follow-up. Progression-free survival (PFS) was defined as the time from surgery to the date of the first recurrence or metastasis. Clinicopathologic variables were entered in the univariate logistic regression analysis. Only variables with a P-value <0.1 were entered into the multivariable logistic regression analysis. Statistical analyses were performed using SPSS 22 software. A 2-sided P<0.05 was considered to be statistically significant.

## Results

### Patient Characteristics

From January 2005 to January 2021, 3191 patients were diagnosed with primary TETs and treated with surgery at Zhongshan Hospital, Fudan University. We excluded patients with incomplete information (153 patients) and those who underwent surgical resection for recurrence (377 patients). A total of 2,561 TET patients remained, and only 81 (2.5%, 81/3191) patients with unresectable TETs received induction therapies followed by surgery. The patient characteristics are presented in **Supplemental Table 1**.

The patients included 45 men and 36 women. At the time of presentation, 52 patients (64.2%) had one or more symptoms, including 19 with chest distress or pain (23.8%), 17 (21.0%) with myasthenia gravis (MG), 9 with cough (11.1%), 4 with fatigue (4.8%), 7 with superior vena cava syndrome (8.6%), and 2 with pure red cell aplastic anaemia (3.2%). The interval time from diagnosis to initial treatment ranged from 0.07 to 24 months (median 1.5). Radiologically, the diameters of TETs ranged from 4.0 cm to 19.0 cm (**Supplemental Figure 1**). The pathological subtypes are shown in **Supplemental Table 1**.

### Treatment Modalities

Treatment strategies of induction therapy were individualized for each patient. The treatment modalities were shown in **Table 1** and **Supplemental Table 2**. Induction therapies consisted of chemotherapy (41 patients, 50.6%), radiotherapy (12 patients, 14.8%), chemoradiotherapy (26 patients, 32.1%), and chemoimmunotherapy (2 patients, 2.5%). When the tumor was considered likely to be incompletely resected, the patients were treated with chemotherapy first. Of the 67 patients receiving chemotherapy, the most commonly used regimen was TP (29 patients, 43.3%). Other cisplatin-based regimens included CAP (15 cases, 22.4%), DP (6 cases, 9.0%), and VP or GP regimens (17 cases, 25.4%). Two patients received chemotherapy (one patient received CAP and one patient received TP) in combination with immunotherapy using camrelizumab. Similar to other malignancies, the addition of radiation to induction chemotherapy has been administered in unresectable TETs with deep intrathoracic or cervical nodal involvement, pleural or pericardial nodules, and pulmonary intraparenchymal nodule or distant organ metastasis. In this study, there were 26 (32.1%) patients received chemo-radiotherapy. Radiation therapy applied as a sole therapeutical induction therapy modality was not routinely suggested in our study. Of these 12 patients receiving induction radiotherapy alone, 8 patients who had huge tumor with pleural, pulmonary or pericardial invasion, but without great vessels invasion, extrathoracic nor intrathoracic metastasis, were deemed not to be a margin-negative surgical resection candidate. Induction radiotherapy was applied for another 4 patients, because they could not tolerate to chemotherapy or were allergic to cytotoxic drug. Induction therapy produced major responses in 56 patients, including 9 (11.1%) complete responses and 47 (58.0%) partial responses with a disease control rate of 95.1%. Pathological R0 resection was performed in 60 patients (74.1%), and 21 patients (25.9%) had incomplete resection. Postoperative pathology revealed that most TETs had improved TRG, with a TRG 1–3 rate of 84.0% (**Supplemental Table 2**).

**Table 1:** Tumor responses and resectability according to induction therapy modalities.

Variables		Cases	Percentage (%)	
Radiotherapy (12 cases)	CR	5	41.7	ORR: 83.3
	PR	5	41.7	
	SD	2	16.7	
	R0	10	83.3	R0: 83.3
	R1	1	8.3	
	R2	1	8.3	
Chemotherapy (41 cases)	CR	1	2.4	ORR: 70.7
	PR	28	68.3	
	SD	9	22.0	
	PD	3	7.3	
	R0	32	78.0	R0: 78.0
	R1	6	14.6	
	R2	3	7.3	
Chemo-radiotherapy (26 cases)	CR	3	11.5	ORR: 61.5
	PR	13	50.0	
	SD	9	34.6	
	PD	1	3.8	
	R0	16	61.5	R0: 61.5
	R1	2	7.7	
	R2	8	30.8	
Chemo-immunotherapy (2 case)	PR	1	–	–
	SD	1	–	
	R0	2	–	–

After patients were evaluated to have recovered from induction therapy (4 to 8 weeks), they underwent surgical resection including the tumor, thymus, mediastinal fat, and all attached structures. However, debulking surgery were considered for thirteen patients to relieve symptoms or reduce tumor burden. Systematic mediastinal lymph node dissection was performed in 23 patients who presented with enlarged lymph nodes, otherwise, only the anterior mediastinal lymph node was resected en bloc with the tumor. Briefly, three different techniques have been suggested to achieve complete resection by reconstruction of the great vessels, including tangential resection and direct suture repair (10 cases), localized resection and repair with pericardial patch (3 cases), and circumferential resection with replacement by polytetrafluoroethylene (PTFE) graft (23 cases). Fifteen patient required pleurectomy for confluent pleural disease. Nine patients received partial sternal resection and chest wall reconstruction. Other structures resected included lung (42 cases), pericardium (32 cases), phrenic nerve (23 cases), brachiocephalic vein (18 cases), diaphragm (10 cases). Twenty-one patients had an incomplete resection, owing to extensive or multi-site infiltration of chest wall (5 cases), main pulmonary artery (2 cases), aorta or arch vessels (7 cases), myocardium (4 cases), trachea (2 cases) or pleural metastasis (13 cases). Nine patients had all gross tumor resected and were thought to be pathological R1 resection and twelve patients had R2 resection.

The decision to use postoperative chemotherapy and/or radiotherapy was individualized for each patient. Briefly, for patients with incomplete resection, adjuvant chemotherapy and/or radiotherapy was performed routinely for a limited time. For those with a close margin or at high risk for recurrence, adjuvant treatment was performed routinely, even if the tumor was completely resected. Postoperative adjuvant treatment was not recommended for patients had complete response and pathological TRG1 with complete resection. Both physiological and psychological conditions of the individual patient were fully taken into account. Adjuvant treatment was not planned for patients receiving induction therapies with maximum tolerated dose of chemotherapy and radiotherapy. Close follow-up without postoperative treatment was also acceptable for patients with intolerance, anaphylaxis or refusing treatment. In this study, thirty-seven patients (45.7%) did not receive postoperative therapy, including 9 cases of TRG1 with R0, 19 cases of maximum tolerated dose of induction chemotherapy and radiotherapy, 4 cases of intolerance or anaphylaxis, and 5 cases of refusing treatment. While, 44 patients (54.3%) underwent postoperative treatment. Detailed information on postoperative treatment is described in **Supplemental Table 2**. Adjuvant therapy was performed in 9 cases of chemotherapy, 23 cases of radiotherapy, 9 cases of chemo-radiotherapy. One patient received postoperative chemotherapy with sintilimab, while two patients received postoperative tyrosine kinase inhibitor (TKI) therapy with surufatinib or anlotinib.

### AEs From Induction Therapy

The common AEs are summarized in **Supplemental Table 3**. The major toxic effects were all-grade haematologic AEs, including anaemia (34.6%) and neutropenia (35.8%). Thirty patients (37.0%) experienced grade ≥ 3 haematologic AEs. The most common all-grade nonhaematologic side effects were weight loss (13.6%) and fatigue (12.3%). Major grade ≥ 3 nonhaematologic toxicities included vomiting (3.7%) and oesophagitis or stomatitis (3.7%). None of the patients had myocardial toxicity or ototoxicity, and no mortalities occurred. The AEs from induction therapy were modest and well tolerated.

### Postoperative Complications and Survival

There were no perioperative deaths in this study. The postoperative length of hospital stay was between 3 and 61 days, with an average of 9.4 ± 7.5 days. There were twelve cases (14.8%) of postoperative complications, including four cases of bleeding, five cases of hydrothorax, two cases of atelectasis and one case of unhealing wounds.

**Figure 1:**
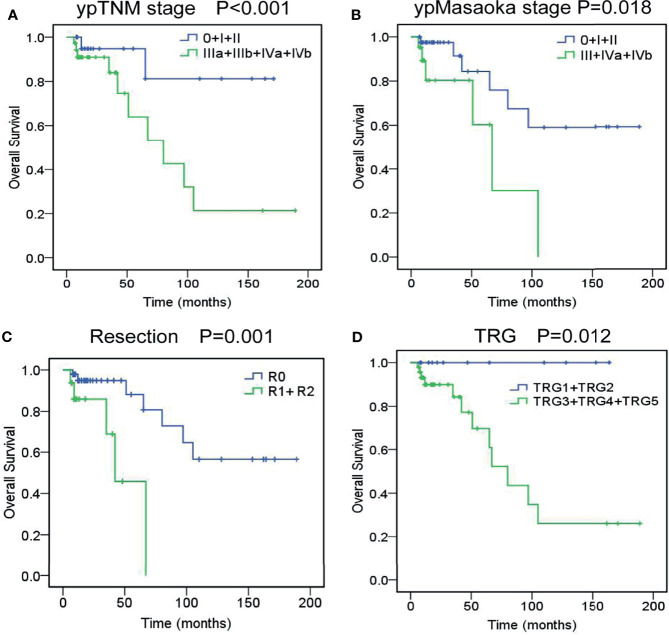
Overall survival curves of unresectable TETs treated with induction therapy followed by surgery, according to ypTNM stage **(A)**, ypMasaoka stage **(B)**, complete resection **(C)**, and tumor response grade **(D)**.

The OS rate was 88.2% at 3 years and 77.8% at 5 years. As shown in **Supplemental Table 4**, univariate analysis revealed that cM stage (P=0.036), cTNM stage (P=0.076), cMasaoka stage (P=0.026), pathological type (P=0.048), ypT stage (P=0.047), ypN stage (P<0.001), ypM stage (P=0.045), ypTNM stage (P<0.001), ypMasaoka stage (P=0.018), complete resection (P=0.001), and TRG (P=0.012) were significant prognostic factors. To rule out confounding factors, we performed multivariate analysis using the Cox proportional hazards model (**Supplemental Table 5**). Multivariate analysis revealed that ypTNM stage (P=0.025), ypMasaoka stage (P=0.031), complete resection (P=0.001), and TRG (P=0.007) were significant independent prognostic factors of OS (**Figure 1**).

**Figure 2:**
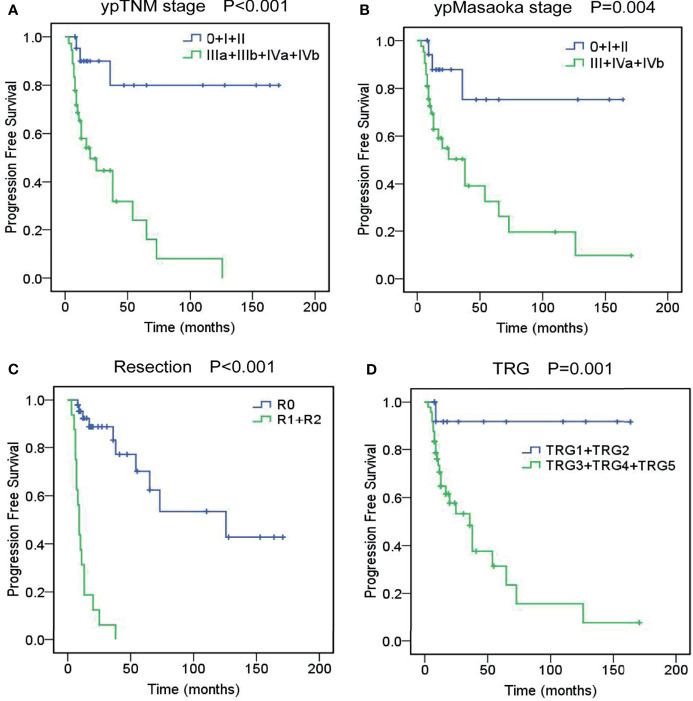
Progression-free survival curves of unresectable TETs treated with induction therapy followed by surgery, according to ypTNM stage **(A)**, ypMasaoka stage **(B)**, complete resection **(C)**, and tumor response grade (TRG) **(D)**.

The 3- and 5-year PFS rates were 58.6% and 46.2%. Univariate analysis revealed that cM stage (P=0.015), cMasaoka stage (P=0.004), pathological type (P=0.033), ypT stage (P<0.001), ypN stage (P<0.001), ypM stage (P<0.001), ypTNM stage (P<0.001), ypMasaoka stage (P=0.004), complete resection (P<0.001), TRG (P=0.001) and postoperative radiotherapy (P=0.018) were significant prognostic factors of PFS. Multivariate analysis showed that ypTNM stage (P=0.001), ypMasaoka stage (P=0.022), complete resection (P<0.001), and TRG (P<0.001) were significant independent prognostic factors of PFS (**Figure 2**).

Survival analyses indicated patients received radical thymectomy with extended resections had similar OS (P=0.065) and PFS (P=0.071) compared to those without extended resection. We also found resected structures were not significant prognostic factors for OS (P=0.136) and PFS (P=0.086), including pericardium, lung, phrenic nerve, chest wall, innomate vein, vena cava, aorta, main pulmonary artery or myocardium.

### Subgroup Analyses of Induction Therapy

In this cohort study, radiotherapy produced the highest objective response rate (ORR) (83.3%) and complete resection rate (83.3%) compared with chemotherapy (ORR: 70.7%, R0: 78.0%) and chemoradiotherapy (ORR: 61.5%, R0: 61.5%) (**Table 1**). Of the 67 patients receiving chemotherapy, the CAP regimen produced the highest ORR (86.7%) and complete resection rate (80.0%) compared with the TP (ORR: 58.6%, R0: 75.9%), DP (ORR: 50.0%, R0: 50.0%), and VP or GP regimens (ORR: 64.7%, R0: 64.7%) (**Table 2**). Patients with thymoma had a better ORR (69.4%) and complete resection rate (75.5%) than those with thymic carcinoma (ORR: 68.9%, R0: 71.9%) (**Table 3**). Induction chemotherapy (P=0.592 for OS, P=0.237 for PFS), induction radiotherapy (P=0.714 for OS, P=0.784 for PFS) and different induction modalities (P=0.645 for OS, P=0.303 for PFS) were not prognostic variables. For the 67 patients receiving chemotherapy, different chemotherapy regimens did not significantly alter the pooled hazard ratio for OS (P=0.887) or PFS (P=0.656) (**Supplemental Table 4**).

**Table 2:** Tumor responses and resectability according to induction chemotherapy regimens.

Variables		Cases	Percentage (%)	
CAP (15 cases)	PR	13	86.7	ORR: 86.7
	SD	2	13.3	
	R0	12	80.0	R0: 80.0
	R1	2	13.3	
	R2	1	6.6	
TP (29 cases)	CR	2	6.9	ORR: 58.6
	PR	15	51.7	
	SD	9	31.0	
	PD	3	10.3	R0: 75.9
	R0	22	75.9	
	R1	2	6.9	
	R2	5	17.2	
DP (6 cases)	PR	3	50.0	ORR: 50.0
	SD	2	33.3	
	PD	1	16.7	
	R0	3	50.0	R0: 50.0
	R1	2	33.3	
	R2	1	16.7	
Other (VP or GP, 17 case)	CR	2	11.8	ORR: 64.7
	PR	9	52.9	
	SD	6	35.3	
	R0	11	64.7	R0: 64.7
	R1	2	11.8	
	R2	4	23.5	

**Table 3:** Tumor responses and resectability according to pathological types.

Variables		Cases	Percentage (%)	
Thymoma (49 cases)	CR	4	8.2	ORR: 69.4
	PR	30	61.2	
	SD	13	26.5	
	PD	2	4.1	
	R0	37	75.5	R0:75.5
	R1	4	8.2	
	R2	8	16.3	
Thymic carcinoma (32 cases)	CR	5	15.6	ORR: 68.9
	PR	17	53.1	
	SD	8	25.0	
	PD	2	6.3	
	R0	23	71.9	R0: 71.9
	R1	6	18.8	
	R2	3	9.4	

### The Values of Clinical and Pathological Downstaging After Induction Therapy

**Figure 3:**
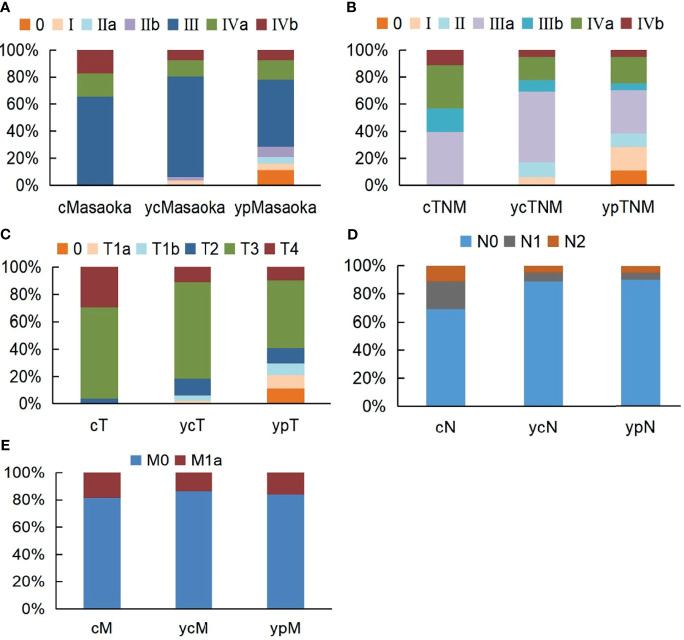
Stage alternations of unresectable TETs treated with induction therapy followed by surgery according to Masaoka **(A)**, TNM **(B)**, T **(C)**, N **(D)**, M **(E)** classifications.

The preinduction treatment clinical (c) stages, postinduction treatment clinical (yc) stages and postoperative pathological (yp) stages are shown in **Figure 3**. Based on the Masaoka stage, clinical downstaging of TETs occurred in 20 patients (24.7%), and pathological downstaging occurred in 35 patients (43.2%). For the TNM staging system, 38 patients (46.9%) had clinical downstaging of disease and 48 patients (59.3%) had pathological downstaging of disease. Exploration research revealed that pathological downstaging of T, N, and M classifications occurred in 42 patients, 18 patients, and 6 patients, respectively. Pathological upstaging was found in 5 patients (6.2%) because of tumour progression or pleural dissemination. Survival analyses indicated that clinical downstaging of the T, N, M, TNM, and Masaoka classifications was not a prognostic factor. Pathological downstaging of the T (P=0.046 for OS, P<0.001 for PFS), N (P=0.003 for OS, P=0.035 for PFS), M (P=0.040 for OS, P=0.048 for PFS), TNM (P=0.018 for OS, P<0.001 for PFS), and Masaoka (P=0.031 for OS, P=0.022 for PFS) classifications had prognostic significance (**Supplemental Table 4**). However, pathological downstaging of neither the TNM classification (P=0.409 for OS, P=0.630 for PFS) nor the Masaoka classification (P=0.708 for OS, P=0.784 for PFS) were independent prognostic variables.

## Discussion

Complete resection is frequently unfeasible in patients with advanced-stage TETs. Induction therapy has been shown to be effective for advanced TETs due to four distinct advantages: (1) producing objective clinical responses and shrinking tumours to relieve symptoms; (2) downstaging tumours and converting unresectable tumours to resectable tumours; (3) improving early local and systemic disease control; and (4) providing evidence of the feasibility and efficacy of drugs or therapies. However, until now, standard induction therapeutic modalities have not been established. Our study further recognizes the clinicopathological features of unresectable TETs and promotes the development of treatment standards and options.

This study was the largest retrospective report to date with favorable long-term outcomes at a single centre. With the use of induction therapies, the R0 rate was 74.1%, with an ORR of 69.1%. These results are in accordance with those of previous studies. In the literature, the range of the ORR in the large series was wide: 62% (13), 73% (14), and 100% (15). The R0 rate reported was also a wide range depending on the study: 22% (16), 43% (13), 69% (15), and 77% (14). The difference among studies might result from the selection of patients, treatment modality, race, and limited number of cases. To date, the recognized results of chemotherapy for TETs have been obtained from the ADOC (doxorubicin, cisplatin, vincristine, cyclophosphamide) regimen, with a 92% ORR and a 43% R0 rate (17). However, which regimens are the best for TETs is unclear thus far. Even worse, direct comparisons between regimens are difficult because of the scarcity of cases and heterogeneity of pathology. Our exploration results showed that the CAP regimen had the highest ORR (86.7%) and R0 rate (80.0%), while the DP regimen had the lowest ORR (50.0%) and R0 rate (50.0%). Although the number of cases was limited, differences in tumour responses and resection among regimens were obvious. The CAP regimen might facilitate complete surgical resection, resulting in a higher cure rate. Discouragingly, our data revealed that the CAP regimen had no survival benefit compared with the other regimens, and different induction chemotherapy regimens did not produce survival differences. Consequently, although this study demonstrated that induction chemotherapy was active against unresectable TETs, we did not definitively draw a conclusion about which regimens offer significant survival advantages.

Based on the European Society of Thoracic Surgeons database, only 1% of TET patients receive radiation alone (18). Induction radiotherapy is an attempt to enhance radical complete resection, but induction radiation has only been reported in small retrospective series. A few studies reported that TET patients treated with preoperative radiation had variable outcomes, with R0 rates ranging from 50% to 75% (5, 18, 19). In this study, 12 of 81 (14.8%) patients received induction radiotherapy alone. We found that induction radiotherapy had a slightly better ORR (83.3%) and R0 rate (83.3%) than chemotherapy. Induction radiation allows more precise radiation planning than postoperative radiation because the initial tumour volume is undisturbed and natural tumour margins can be dimensioned without disturbance. Our data implied that radiation had an important role in the local control of TETs. However, there were no differences in OS and PFS between patients who received induction radiation and those who did not. Our data shed light on the rationale for induction radiotherapy in unresectable TET patients.

The combination of induction radiation and chemotherapy has been used in TETs, with a wide range of ORRs (40%-70%) and R0 rates (60%-80%) (4, 6, 20). All patients with unresectable TETs received multidisciplinary consultation for mediastinal tumours in our centre. In this series, 26 patients (32.1%) received chemoradiotherapy, with an ORR of 61.5% and an R0 rate of 61.5%. This retrospective study demonstrated that chemoradiotherapy did not achieve better tumour control and surgical resection rates than chemotherapy or radiotherapy alone. Unsurprisingly, we also found that chemoradiotherapy did not offer a survival benefit compared with chemotherapy or radiotherapy alone. Induction therapy facilitates tumour control and surgical removal. However, induction strategies need further research to achieve successful treatment of unresectable TETs. The tricky problem of induction therapy is the toxicity of induction. In the current study, our results demonstrated that AEs from induction therapy were modest and well tolerated. The efficacy and safety of induction therapy should be established by randomized trials to essentially decide optimal treatment.

Theoretically, the potential benefit of induction therapy is the downstaging of bulky tumours. In fact, clinical Masaoka downstaging only occurred in 24.7% of patients, and 46.9% of patients had clinical TNM downstaging. Our results seemed to be in line with the low clinical downstaging rate in a previous report by Park et al. (21) Moreover, we found that patients who were clinically downstaged after induction therapy had similar clinical outcomes compared with those who were not. Most importantly, many variations make it difficult to evaluate clinical stage alterations, such as research subjectivity, observation of lymph node metastasis, and measurement bias of tumour size. We analyzed T, N, M and TNM classifications to provide granularity of tumour stages. Our data showed that clinical and pathological downstaging of the Masaoka, TNM, T, N, or M classifications were not independent prognostic variables. Pathological examination is the gold standard to assess tumour downstaging from induction therapy. Consequently, clinical downstaging could be underestimated and has no clear survival implications for unresectable TETs.

Only a few studies have reviewed the histologic response of TETs to induction treatment (11). We classified a reproducible five-tier TRG system for TET specimens after induction treatment. Previous studies reported the percentage of viable tumours after preoperative therapy for both resectable and unresectable TETs. The results were quite variable, with 0% to 100% viable tumour cells (11, 13, 15, 20). The cause for pathological response differences is not entirely clear but could be due to diverse induction treatment modalities. In this study, we found that the morphological response to induction treatment correlated with TRG. All patients with TRG 1 had a complete radiologic response, whereas those with TRG 5 did not show any radiologic response but had tumour progression. Moreover, patients with radiologic PR had a better TRG and a lower percentage of viable tumours than those with SD. Given the close correlation of radiologic response and TRG, we selected TRG as a prognostic variable in survival analyses. TRG was not only a prognostic factor but also a significant independent prognostic factor for OS and PFS by multivariate analyses. The completeness of resection significantly increases the survival time, even for primary unresectable TETs. Among various clinical factors, R0 resection was the most important independent prognostic factor of OS and PFS for unresectable TETs. However, tumour regression does not guarantee the achievement of R0 resection. In our study, the incomplete resection rate was 25.9%, although only 13 patients (16.0%) had TRG 4–5. The success of R0 resection depends on whether involved structures can be removed or reconstructed. R0 resection is our primary goal, and every effort should be made to achieve resectability.

There were considerable challenges in this study. First, the data were retrospectively reviewed from a single centre. Thus, there was inevitable selection bias. Second, a wide variety of induction therapies were used in our patients. Chemotherapy regimens were not the uniform protocol owing to heterogeneous histological subtypes. Third, although this study gave a rough indication of distinct responses to different induction therapies, direct comparisons between regimens were limited. Given the rarity of the tumour, enrolling a large number of patients with complete survival data will unavoidably take many years. To establish a more effective formula for induction therapy, multicentre randomized controlled trials with the best supportive care are necessary.

## Conclusion

In this study, we retrieved 81 patients with unresectable TETs received induction therapy followed by surgery. We found induction therapy produced major tumor responses of 69.1% and tumor response grade (TRG) 1-3 of 84.0%. Moreover, R0 resection rate was 74.1% with the 10-year OS of 45.7% and 10-year PFS of 35.2%. We also found ypTNM stage, ypMasaoka stage, complete resection, and TRG were all independent significant prognostic factors. Exploratory research revealed different induction modalities and different chemotherapy regimens had no significant survival difference. Neither clinical nor pathological downstaging of T, N, M, TNM, Masaoka classifications were independent prognostic variables. Therefore, induction therapy followed by surgery provides good control of tumor for unresectable TETs patients.

## Data Availability Statement

The raw data supporting the conclusions of this article will be made available by the authors, without undue reservation.

## Ethics Statement

Approval was obtained from the Research Ethics Committee of Zhongshan Hospital, Fudan University (Y2019-187).

## Author Contributions

SW and JIAD conceived and supervised the research. SW, JJ, GC, YF, BX, JIAD, SD, JL, and JIHD contributed to the design of the project and discussions. JJ, SW, and JIAD contributed to data analyses; SW, JJ, GC, YF, BX, JIHD, SD, JL, and JIAD contributed to materials or clinical data; SW, JJ, and JIAD wrote the manuscript. All authors contributed to the article and approved the submitted version.

## Funding

This work was supported by grants from the National Natural Science Foundation of China (81972168).

## Conflict of Interest

The authors declare that the research was conducted in the absence of any commercial or financial relationships that could be construed as a potential conflict of interest.

## Publisher’s Note

All claims expressed in this article are solely those of the authors and do not necessarily represent those of their affiliated organizations, or those of the publisher, the editors and the reviewers. Any product that may be evaluated in this article, or claim that may be made by its manufacturer, is not guaranteed or endorsed by the publisher.
