# Supplementary material for: Induction Therapy Followed by Surgery for Unresectable Thymic Epithelial Tumours
Source: Front Oncol. 2022 Jan 5;11:791647. doi: 10.3389/fonc.2021.791647 (PMC8766658; doi:10.3389/fonc.2021.791647)

Supplemental Figure 1. CT images of unresectable TETs patients treated with induction therapy followed by surgery. A: stable disease to induction therapy, B: partial response to induction therapy.

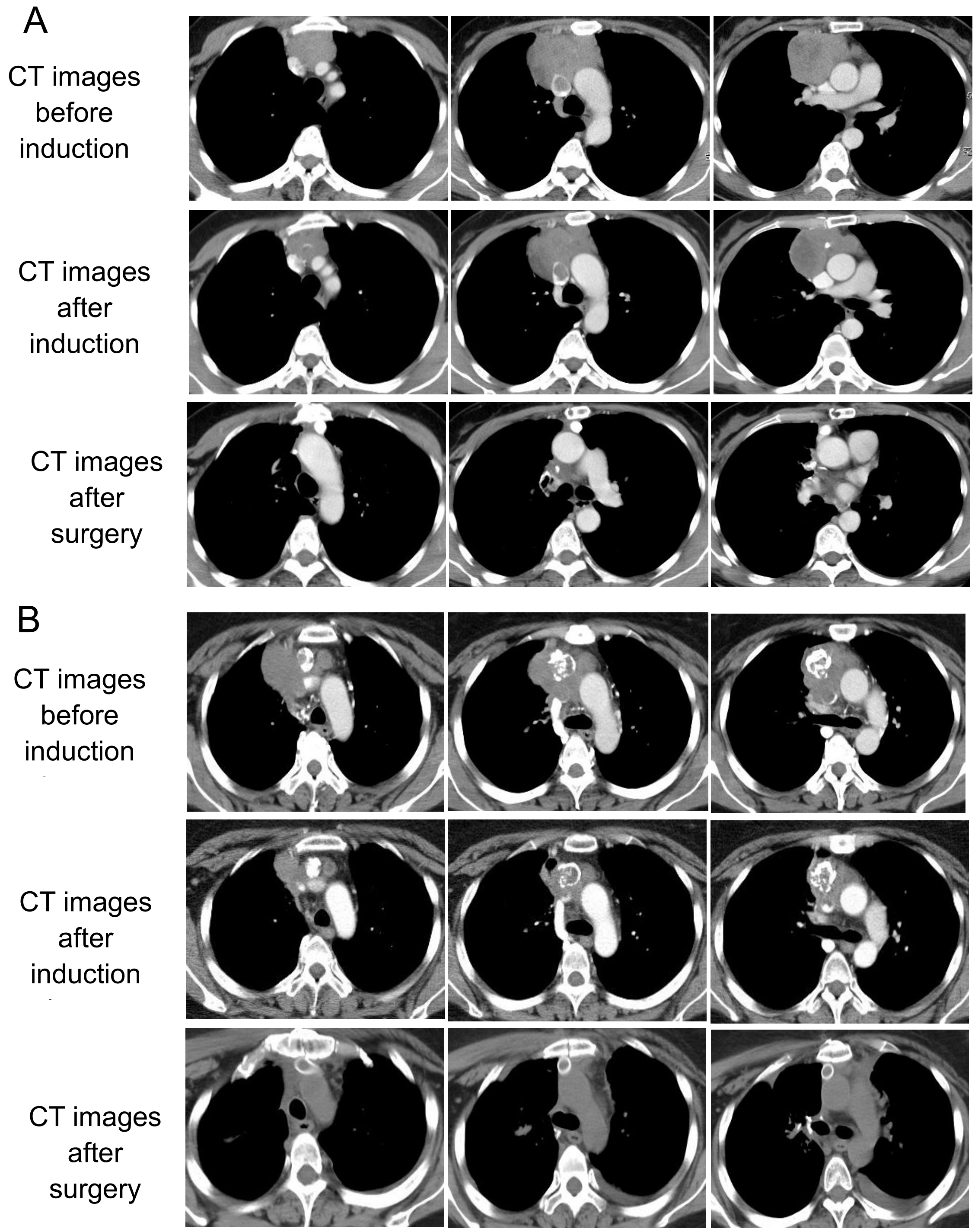

Supplement: Supplementary file 1 [file DataSheet_1.pdf]
