# Supplementary material for: Induction Therapy Followed by Surgery for Unresectable Thymic Epithelial Tumours
Source: Front Oncol. 2022 Jan 5;11:791647. doi: 10.3389/fonc.2021.791647 (PMC8766658; doi:10.3389/fonc.2021.791647)
Supplement: Supplementary file 2 [file Table_1.docx]

**Supplemental Table 1**. Characteristics of 81 Patients with Unresectable TETs Treated with Induction Therapy Followed by Surgery.

| Variables | | | Cases | Percentage (%) |
| --- | --- | --- | --- | --- |
| Gender | | Male | 45 | 55.6 |
|  |  | Female | 36 | 44.4 |
| Age | | ≧60Y | 15 | 18.5 |
|  |  | <60Y | 66 | 81.5 |
| sympotom | | Yes | 52 | 64.2 |
|  |  | No | 29 | 35.8 |
| Time interval | | ≧3 months | 40 | 49.4 |
|  |  | <3 months | 41 | 50.6 |
| Period | | 2005-2017 | 21 | 25.9 |
|  |  | 2018-2021 | 60 | 74.1 |
| Tumor size | | ≧ 8cm | 37 | 45.7 |
|  |  | < 8cm | 44 | 54.3 |
| Pathology | Thymoma  (49) | A | 5 | 6.2 |
|  |  | AB | 3 | 3.7 |
|  |  | B1 | 3 | 3.7 |
|  |  | B2 | 18 | 22.2 |
|  |  | B3 | 13 | 16.1 |
|  |  | B2+B3 | 7 | 8.6 |
|  | Thymic carcinoma  (32) | Squamous | 20 | 24.7 |
|  |  | Adenocarcinoma | 2 | 2.5 |
|  |  | Carcinoid | 1 | 1.2 |
|  |  | Small cell | 1 | 1.2 |
|  |  | Large-cell | 2 | 2.5 |
|  |  | Basaloid | 1 | 1.2 |
|  |  | Lymphoepithelioma-  like | 1 | 1.2 |
|  |  | Undifferentiated | 4 | 4.9 |
