# Supplementary material for: Induction Therapy Followed by Surgery for Unresectable Thymic Epithelial Tumours
Source: Front Oncol. 2022 Jan 5;11:791647. doi: 10.3389/fonc.2021.791647 (PMC8766658; doi:10.3389/fonc.2021.791647)
Supplement: Supplementary file 3 [file Table_2.docx]

**Supplemental Table 2**. Treatment modalities and tumor responses of unresectable TETs treated with induction therapy followed by surgery.

| Variables | | Cases | Percentage  (%) |
| --- | --- | --- | --- |
| Induction  therapy | Chemotherapy | 41 | 50.6 |
|  | Radiotherapy | 12 | 14.8 |
|  | Chemo-radiotherapy | 26 | 32.1 |
|  | Chemo-immunotherapy | 2 | 2.5 |
| Tumor  response | CR | 9 | 11.1 |
|  | PR | 47 | 58.0 |
|  | SD | 21 | 25.9 |
|  | PD | 4 | 4.9 |
| Resection | R0 | 60 | 74.1 |
|  | R1 | 9 | 11.1 |
|  | R2 | 12 | 14.8 |
| Tumor response grade | TRG1 | 9 | 11.1 |
|  | TRG2 | 11 | 13.6 |
|  | TRG3 | 48 | 59.3 |
|  | TRG4 | 9 | 11.1 |
|  | TRG5 | 4 | 4.9 |
| POD (3-61 Days) | ≧ 7 | 37 | 45.7 |
|  | < 7 | 44 | 54.3 |
| Postoperative  complications | Yes | 12 | 14.8 |
|  | No | 69 | 85.2 |
| Postoperative  therapy | Chemotherapy | 9 | 11.1 |
|  | Radiotherapy | 23 | 28.4 |
|  | Chemo-radiotherapy | 9 | 11.1 |
|  | TKI  or  Immunotherapy | 3 | 3.7 |
|  | None | 37 | 45.7 |
