# Supplementary material for: Induction Therapy Followed by Surgery for Unresectable Thymic Epithelial Tumours
Source: Front Oncol. 2022 Jan 5;11:791647. doi: 10.3389/fonc.2021.791647 (PMC8766658; doi:10.3389/fonc.2021.791647)
Supplement: Supplementary file 4 [file Table_3.doc]

**Supplemental Table 3.** Adverse events of induction therapy for unresectable TETs.

| Adverse events | | All grades | | Grade ≥ 3 | |
| --- | --- | --- | --- | --- | --- |
|  |  | Cases | Percentage (%) | Cases | Percentage (%) |
| Hematologic | Anemia | 28 | 34.6 | 11 | 13.6 |
|  | Neutropenia | 29 | 35.8 | 13 | 16.0 |
|  | Thrombocytopenia | 9 | 11.1 | 4 | 4.9 |
|  | Myelosuppression | 6 | 7.4 | 2 | 2.5 |
| Non-  hematologic | Fatigue | 10 | 12.3 | 0 | -- |
|  | Nausea | 9 | 11.1 | 0 | -- |
|  | Vomiting | 8 | 9.9 | 3 | 3.7 |
|  | Myalgia | 7 | 8.6 | 0 | -- |
|  | Alopecia | 5 | 6.2 | 0 | -- |
|  | Esophagitis or  Stomatitis | 9 | 11.1 | 3 | 3.7 |
|  | Diarrhea | 4 | 4.9 | 0 | -- |
|  | Pneumonia | 7 | 8.6 | 2 | 2.5 |
|  | Peripheral  neuropathy | 2 | 2.5 | 0 | -- |
|  | Weight loss | 11 | 13.6 | 0 | -- |
