# Supplementary material for: Induction Therapy Followed by Surgery for Unresectable Thymic Epithelial Tumours
Source: Front Oncol. 2022 Jan 5;11:791647. doi: 10.3389/fonc.2021.791647 (PMC8766658; doi:10.3389/fonc.2021.791647)
Supplement: Supplementary file 5 [file Table_4.doc]

**Supplemental Table 4.** Univariate analyses of survival for unresectable TETs treated with induction therapy followed by surgery.

| Variable | OS | |  | PFS | |
| --- | --- | --- | --- | --- | --- |
|  | Chi-Square | P |  | Chi-Square | P |
| Age  < 50yrs vs. ≥50 yrs | 0.398 | 0.528 |  | 0.276 | 0.601 |
| Gender  Male vs. Female | 1.171 | 0.279 |  | 2.502 | 0.114 |
| Period  2006-2017 vs. 2018-2021 | 0.268 | 0.605 |  | 1.649 | 0.199 |
| Symptom  Yes vs. No | 2.557 | 0.110 |  | 1.395 | 0.211 |
| Interval time  ≤1.5 vs. >1.5 months | 1.452 | 0.228 |  | 0.340 | 0.560 |
| Tumor size  ≤8 vs. >8 cm | 0.045 | 0.833 |  | 0.505 | 0.477 |
| Clinical T stage  cT2+cT3 vs. cT4 | 1.486 | 0.223 |  | 0.209 | 0.648 |
| Clinical N stage  cN0 vs. cN1+cN2 | 3.354 | 0.067 |  | 3.619 | 0.051 |
| Clinical M stage  cM0 vs. cM1a | 3.925 | 0.036 |  | 5.898 | 0.015 |
| Clinical TNM stage  cIIIa vs. cⅢb+cⅣa+cⅣb | 3.269 | 0.076 |  | 3.556 | 0.054 |
| Clinical Masaoka stage  cIII vs. cⅣa+cⅣb | 4.987 | 0.026 |  | 8.091 | 0.004 |
| Induction chemotherapy  Yes vs. No | 0.287 | 0.592 |  | 1.401 | 0.237 |
| Induction chemotherapy regimen  CAP vs. TP vs. DP vs. GP+VP | 1.140 | 0.887 |  | 2.437 | 0.656 |
| Induction radiotherapy  Yes vs. No | 0.135 | 0.714 |  | 0.075 | 0.784 |
| Induction therapy  Chemotherapy vs. Radiotherapy  vs. Chemo-rediotherapy | 0.878 | 0.645 |  | 2.388 | 0.303 |
| Clinical T downstage  Yes vs. No | 0.211 | 0.646 |  | 0.424 | 0.515 |
| Clinical N downstage  Yes vs. No | 0.003 | 0.958 |  | 0.226 | 0.635 |
| Clinical M downstage  Yes vs. No | 0.096 | 0.757 |  | 0.720 | 0.396 |
| Clinical TNM downstage  Yes vs. No | 0.479 | 0.489 |  | 0.862 | 0.353 |
| Clinical Masaoka downstage  Yes vs. No | 0.689 | 0.407 |  | 1.397 | 0.237 |
| Pathological T downstage  Yes vs. No | 3.701 | 0.046 |  | 14.369 | <0.001 |
| Pathological N downstage  Yes vs. No | 8.301 | 0.003 |  | 3.726 | 0.035 |
| Pathological M downstage  Yes vs. No | 3.746 | 0.040 |  | 3.655 | 0.048 |
| Pathological TNM downstage  Yes vs. No | 5.615 | 0.018 |  | 13.753 | <0.001 |
| Pathological Masaoka downstage  Yes vs. No | 3.876 | 0.031 |  | 5.223 | 0.022 |
| Pathological type  Thymoma vs. carcinoma | 3.654 | 0.048 |  | 3.788 | 0.033 |
| Pathological T stage  ypT0+ypT1+ypT2 vs. ypT3+ypT4 | 3.686 | 0.047 |  | 12.648 | <0.001 |
| Pathological N stage  ypN0 vs. ypN1+ ypN2 | 14.642 | <0.001 |  | 15.285 | <0.001 |
| Pathological M stage  ypM0 vs. ypM1a | 3.524 | 0.045 |  | 15.269 | <0.001 |
| Pathological TNM stage  yp0+ypI+ypII vs. ypⅢa+  ypⅢb+ypⅣa+ypⅣb | 15.141 | <0.001 |  | 16.697 | <0.001 |
| Pathological Masaoka stage  yp0+ypI+ypII vs. ypⅢ+  ypⅣa+ypⅣb | 6.170 | 0.018 |  | 8.496 | 0.004 |
| Resection  R0 vs. R1+R2 | 10.131 | 0.001 |  | 53.753 | <0.001 |
| TRG  TRG1+TRG2 vs. TRG3+  TRG4+TRG5 | 6.274 | 0.012 |  | 10.360 | 0.001 |
| Postoperative chemotherapy  Yes vs. No | 1.349 | 0.245 |  | 3.631 | 0.057 |
| Postoperative radiotherapy  Yes vs. No | 0.379 | 0.538 |  | 5.640 | 0.018 |
| Postoperative therapy  None vs. Chemotherapy vs. Radiotherapy vs. Chemo-radiotherapy | 3.703 | 0.295 |  | 6.039 | 0.171 |
| Extended resection  Yes vs,No | 3.028 | 0.065 |  | 3.061 | 0.071 |
| Postoperative therapy  None vs. Chemotherapy vs. Radiotherapy vs. Chemo-radiotherapy | 3.703 | 0.295 |  | 6.039 | 0.171 |
