# Supplementary material for: Induction Therapy Followed by Surgery for Unresectable Thymic Epithelial Tumours
Source: Front Oncol. 2022 Jan 5;11:791647. doi: 10.3389/fonc.2021.791647 (PMC8766658; doi:10.3389/fonc.2021.791647)
Supplement: Supplementary file 6 [file Table_5.doc]

**Supplemental Table 5.** Multivariate Survival Analyses of 81 Patients with Unresectable TETs Treated with Induction Therapy Followed by Surgery.

| Variable | OS | |  | PFS | |
| --- | --- | --- | --- | --- | --- |
|  | HR (95% CI) | P |  | HR (95% CI) | P |
| cTNM stage  Ⅲb+Ⅳa+Ⅳb vs. IIIa | 1.820  (0.074-9.135) | 0.872 |  | 1.270  (0.282-5.723) | 0.756 |
| cMasaoka stage  Ⅳa+Ⅳb vs. III | 1.771  (0.061-9.717) | 0.840 |  | 1.860  (0.199-3.742) | 0.841 |
| Pathological TNM downstage  No vs. Yes | 3.035  (0.218-10.284) | 0.409 |  | 1.637  (0.101-4.002) | 0.630 |
| Pathological Masaoka downstage  No vs. Yes | 1.557  (0.026-11.929) | 0.708 |  | 1.749  (0.095-5.909) | 0.784 |
| Pathological type  Carcinoma vs. thymoma | 1.443  (0.103-1.918) | 0.276 |  | 1.823  (0.315-1.312) | 0.690 |
| ypTNM stage  Ⅲa+Ⅲb+Ⅳa+Ⅳb vs. 0+I+II | 1.903  (1.017-2.584) | 0.025 |  | 2.0475  (1.164-2.996) | 0.010 |
| ypMasaoka stage  Ⅲ+Ⅳa+Ⅳb vs. 0+I+II | 1.837  (1.104-2.691) | 0.031 |  | 1.843  (1.067-2.982) | 0.022 |
| Resection  R1+R2 vs. R0 | 2.485  (1.638-3.722) | 0.001 |  | 3.282  (1.922-5.396) | <0.001 |
| TRG  TRG 3-5 vs. TRG 1-2 | 2.143  (1.163-3.561) | 0.007 |  | 3.037  (1.819-4.875) | <0.001 |
| Postoperative chemotherapy  No vs. Yes | -- | -- |  | 1.810  (0.501-1.312) | 0.392 |
| Postoperative radiotherapy  No vs. Yes | -- | -- |  | 1.487  (0.829-2.668) | 0.183 |
